# Inefficiencies of augmented reality for different sexes and grades in Chinese vocational education

**DOI:** 10.1038/s41598-023-48727-9

**Published:** 2023-12-02

**Authors:** Mingjian Yang, Dandan She, Qiong Xu, Wei Zhang, Chaonan Qu, Xiaofei Hu

**Affiliations:** 1 School of Architecture and Engineering, Jiujiang Vocational and Technical College, Jiujiang, 332007 China; 2 Department of Foreign Language Teaching, Jiujiang Vocational and Technical College, Jiujiang, 332007 China; 3 School of Finance and Accounting, Jiujiang Vocational and Technical College, Jiujiang, 332007 China; 4 School of Psychology, Shaanxi Normal University, Xi’an, 710062 China

**Keywords:** Psychology, Human behaviour

## Abstract

Nowadays, augmented reality (AR) is becoming more and more prevalent and used in many fields, including education. Previous studies have reported the positive effect of AR to advocate the use of AR in the classroom. However, it is unclear whether such a positive effect can be reported for any student. In this study, we recruited students from a Chinese vocational college. Due to the peculiar conditions in China, students in vocational colleges may have weaker learning abilities and worse academic performance than students in research universities. Furthermore, the sex and grade of students were considered. We conducted a three-stage experiment for the PPT-based group and the AR-based group separately. We taught the students the orthographic projection, a lesson from the Engineering Drawing course, which is widely learned by students who majored in architecture. The students’ academic performances were rated prior to class, right after class, and 1-week after class, through a direct examination at three stages. We found the inefficiency of AR for students in vocational colleges. The interaction effect between sex and grade of students was also reported. Students undergoing AR-based teaching could not perform better or even worse than students undergoing PPT-based teaching. We recommended that the teachers should consider whether to use AR in the classroom based on the characteristics of students.

## Introduction

In the novel “The Master Key” written by Lyman Frank Baum, the main character acquires the supernatural power of “character mark”, which can see the potential character letters on other people’s foreheads through special eyes, creating the illusion of a modern-like technology, that is, the augmented reality (AR). Azuma^1^ defines AR as a technology that enhances the users’ perception of the real world by combining the upper and lower layers of digital information with the real world. AR can show almost everything at any time to provide concrete images for users at less expense. Due to the development of mobile and rendering technologies, AR has gained wide application in many fields, such as medical training, tourism, manufacturing, marketing, architecture, and education^1–12^. The enormous applications on your mobile phones are evidence of AR’s prevalence.

In the current study, we focused on the educational field. By combining the media features, such as sensory immersion, with traditional teaching, AR could positively contribute to students’ affective states, thus enhancing learning efficiency^11,13,14^. For example, Di Serio et al.^15^ reported the positive effect of AR on the motivation of secondary school students. They found that the motivation of middle-school students was higher in an AR-based learning environment than those obtained in a slide-based learning environment. Furthermore, researchers designed a system that enhances the AR experience in the classroom^2,16^. For example, El Sayed et al.^16^ designed an AR student cards system to increase visualization ability without using too many tools in the classroom. Taken together, it becomes more and more prevalent to deploy AR in the classroom.

However, as the receiver of a normal class, students were merely discussed in the previous studies. According to an elegant review of AR in education, previous studies mainly focused on science domains, such as physics, life sciences, and geometry, for middle school students and undergraduate students in research universities^14^. The main task of such a class was to help students acquire theoretical knowledge. On the other hand, few studies recruited students in vocational colleges to examine whether AR positively affected the learning process^2,17^. The main task of the vocational college is to help students acquire practical skill training and hands-on learning ability with the least fundamental knowledge. For example, students in vocational colleges were trained through the use of the LabVIEW virtual instrument program^18^. Ortega-Gras et al.^19^ developed immersive extended reality training content to teach students in vocational colleges in the circular wood and furniture sector. Windelband^20^ summarized the opportunities and risks of artificial intelligence and assistance systems for vocational education, claiming the prevalence of new technology in vocational training nowadays. Vocational education and research education exhibit distinct differences. Research education emphasizes the enhancement of theoretical knowledge, as manifested in various exercises such as computational problems, fill-in-the-blanks, and multiple-choice questions; however, vocational education places a premium on practical hands-on skills, for example, the speed and accuracy of instrument installation or drafting plans. Vocational education caters to positions requiring technical skills, marking a fundamental difference from research education. Therefore, different educational institutions have distinct aims in teaching, which in turn necessitates different methodologies for appraising academic proficiency. For students in vocational colleges, evaluations ought to lean towards more applicable, practical assays to accurately assess their academic performance. Moreover, it is worth noting that vocational college in China has peculiar property. The requirements for enrollment in research and vocational colleges are extremely different, with higher scores in the entrance examination for the former and lower scores for the latter, leading to different learning abilities for students in different types of universities. Students from vocational colleges tended to have lower scores in digital competencies, which were defined as the ability to use digital technologies in the academic fields, than students in research universities^21,22^. In a study wherein second-year students who were trained to be a teacher were recruited as the participants, the utilization of AR technology did not improve the perceived usefulness and future applicability of AR among future teachers^23^. Therefore, it doubted whether the positive effect of AR could be reported for students in vocational colleges with different teaching objectives and learning abilities.

Besides, the demographic characteristics of students were not discussed in the previous studies. The first characteristic was the nationality of students. Since we recruited students from China, this would not be discussed. The second characteristic was the sex of the students. There was some evidence of the sex difference when using new technology in the classroom, such as online courses^24,25^. For example, males tended to use the Internet more frequently and intensely than females^25^. Male students were more positive about interactive systems in the classroom than female students^24^. It is possible that male students can outperform female students when learning with AR because of males’ stronger intention to use new technology^26^. However, other studies proposed a different possibility. Females were more likely to use the Internet for study-related activities than males^27^. Female students were more motivated, had a stronger desire to study, and were more self-regulated than male students^28–30^. It is also possible that female students can outperform male students because of females’ willingness to study online^31^. Considering the inconsistency among previous studies, it is necessary to examine whether there was a sex difference in the effect of AR on students’ academic performance.

The third characteristic is the age of students. By conducting a set of 25 tasks developed based on the European Digital Competence framework DigComp 2.1., Barboutidis and Stiakakis^22^ reported that participants between 36 and 45 years old differed significantly from those between 18 and 25 years old in the “Communication and collaboration” area of DigComp. For students in vocational colleges, their ages are converged within the range of 18–20 years old. It is not very meaningful to discuss the biological age of students. Instead, the grade of students (student’s year of study at university) might be an important factor that could affect academic performance. Previous studies have reported many differences among students with different grades. For example, freshmen and sophomores were more likely to ask for help from peers and teachers and relate class material to their future careers than juniors and seniors^32,33^. Juniors and seniors might be good at time management and spend more time coping with schoolwork, socializing, and future career^34^. Seniors had a more positive attitude toward internet-aided instruction than freshmen, sophomores, and juniors^35,36^. Freshmen and sophomores tended to feel more depressed and stressed than seniors^37^. Considering the different abilities, learning strategies, attitudes, and psychological states among students with different grades, it is necessary to examine whether there was a grade difference in the effect of AR on students’ academic performance.

To sum up, we conducted a between-subject experiment to examine the effect of AR on students’ academic performance. Specifically, we focused on the students in Chinese vocational colleges with different demographic characteristics (sex and grade). The recruited students were distributed into two teaching groups: PPT-based and AR-based. Within each group, the sex and grade of students were further regarded as between-subject factors. We aimed to answer two questions: can the utilization of AR technology in Chinese vocational colleges improve the academic performance of students, and do the demographic characteristics (sex and grade) of students affect the effect of the utilization of AR technology? It is true that students in vocational colleges are generally considered to have poor learning abilities and are trained to be a doer, not a thinker, in China. However, according to previous studies, we hypothesized that students’ academic performance could be greatly improved due to the vivid presentation of learning materials, and this elevation should not be affected by the sex and grade of students. In the current study, we utilized a portion of the Engineering Drawing course as a method to assess the academic performance of students. Students were asked to draw the frontal view, side view, and top view of a 3D material. The course of Engineering Drawing is commonly offered and has wide popularity. It is a fundamental course for students in science and engineering disciplines. It equips students with the ability to communicate their ideas and designs visually and effectively, and it’s an essential skill for a wide range of professions, including architects, engineers, and designers. The course typically involves learning how to draw using CAD (Computer-Aided Design) software, as well as learning the fundamental principles of design and drafting. We chose this course due to its high degree of practicality, which can better reflect the teaching objectives of students in vocational colleges. We rated students’ scores at three test times to track their academic performances through a direct examination: prior to class, right after class, and 1-week after class.

## Results

**Figure 1:**
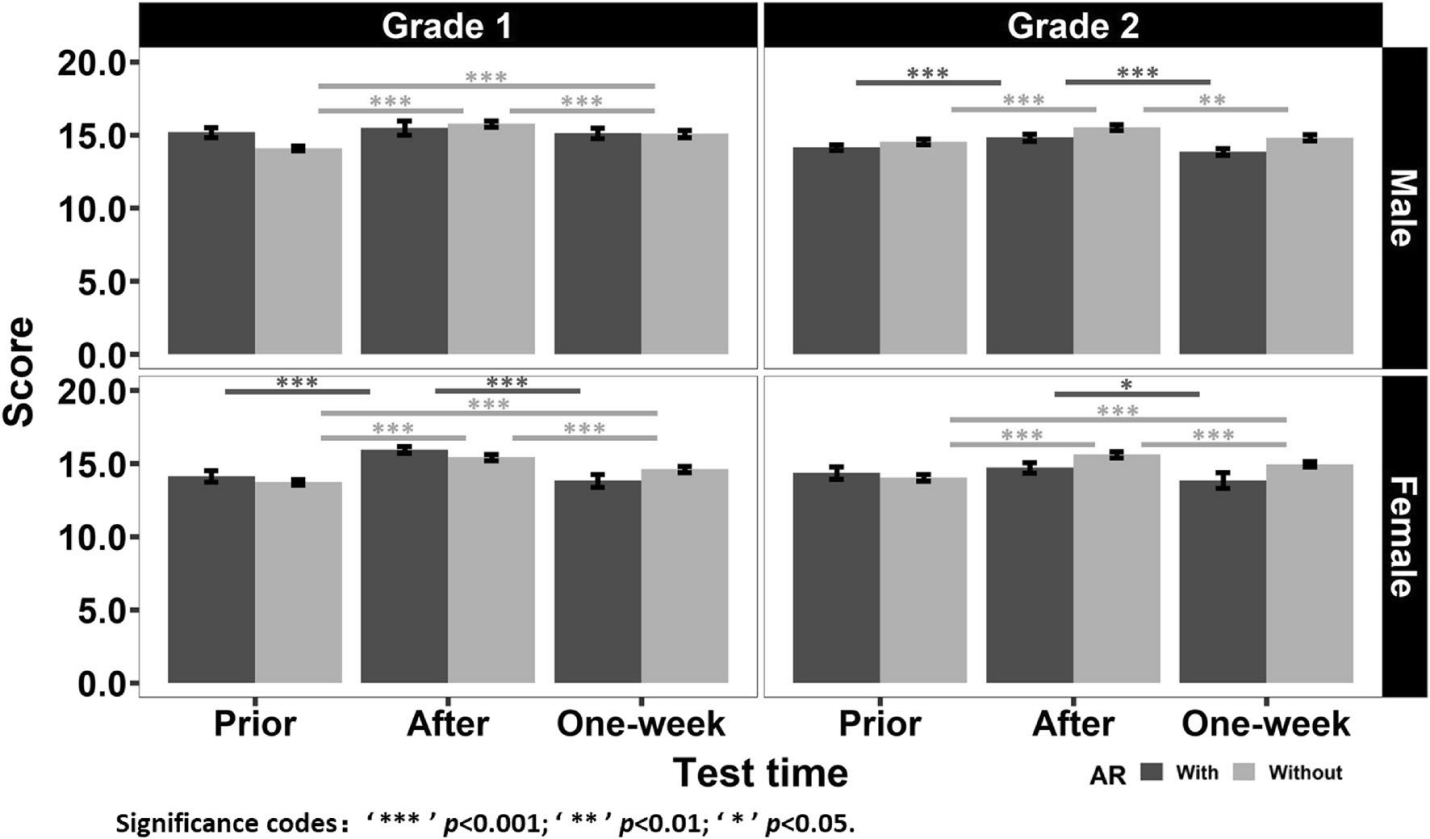
The results of scores. The left and right panels represent the results for students with different grades, and the top and bottom panels represent the results for students of different sexes. Within each panel, the abscissa represents the test time, and the ordinate represents the average scores. The color of the bars represents whether the students learned with AR or not. The lines on the top of the bars represent the post-hoc results, with the color of the lines corresponding to that of the bars. The error bars represent the standard error of the means across students.

The results of the scores are depicted in Fig. 1. The results for students with different sexes and grades were summarized into different panels, within which the color of the bars represents the AR condition. We performed a four-way mixed analysis of variance (ANOVA) on the scores to examine what factors could affect the scores. The within-subject factors included the test time (prior to class, right after class, and 1-week after class). The between-subject factors included sex (male and female), grade (grade 1 and grade 2), and AR condition (with AR and without AR). The sphericity was corrected by the Greenhouse-Geisser method if the within-subject factor violated the sphericity assumption, with the Greenhouse-Geisser epsilon (GGe) being 0.862. We found the significant main effect for test time ($F(1.72,327.72) = 100.702, p < 0.001, \eta _p^2 = 0.346$), whereas all the remaining main effects were not significant ($p > 0.05$). We also found the significant two-factor interaction effects between grade and AR condition ($F(1,190) = 4.869, p = 0.029, \eta _p^2 = 0.025$), sex and test time ($F(1.72,327.72) = 4.571, p = 0.015, \eta _p^2 = 0.023$), grade and test time ($F(1.72,327.72) = 4.189, p = 0.021, \eta _p^2 = 0.022$), AR condition and test time ($F(1.72,327.72) = 20.388, p < 0.001, \eta _p^2 = 0.097$). Furthermore, the three-factor interaction effect among sex, grade, and test time ($F(1.72,327.72) = 4.729, p = 0.013, \eta _p^2 = 0.024$), and the four-factor interaction effect ($F(1.72,327.72) = 7.046, p = 0.002, \eta _p^2 = 0.036$) were significant. All the remaining interaction effects were non-significant ($p > 0.05$). The descriptive results of scores for students with different sexes and grades are summarized in Table 1.

**Table 1:** The descriptive results of scores for students with different sexes and grades. The standard errors are placed in parentheses.

	Prior		After		One-week	
	Grade 1	Grade 2	Grade 1	Grade 2	Grade 1	Grade 2
Male						
With	15.175 (0.343)	14.140 (0.197)	15.475 (0.486)	14.816 (0.237)	15.125 (0.358)	13.844 (0.225)
Without	14.062 (0.163)	14.538 (0.190)	15.759 (0.203)	15.510 (0.200)	15.076 (0.235)	14.807 (0.223)
Female						
With	14.122 (0.385)	14.357 (0.427)	15.944 (0.214)	14.700 (0.362)	13.822 (0.432)	13.843 (0.544)
Without	13.725 (0.171)	14.038 (0.227)	15.413 (0.223)	15.615 (0.214)	14.600 (0.222)	14.954 (0.174)

Considering the objective of the current study, wherein we aimed to examine whether students’ academic performance could be improved with AR and the fact that all four factors interacted significantly, we then performed a two-way mixed ANOVA, with AR condition regarded as the between-subject factor and test time regarded as the within-subject factor, for students with different sexes and grades separately.

### Separate analyses for different sexes and grades

For the grade 1 and male students (top-left panel of Fig. 1, GGe = 0.759), the main effect for test time ($F(1.52,65.27) = 13.438, p < 0.001, \eta _p^2 = 0.238$) and the interaction effect ($F(1.52,65.27) = 7.172, p = 0.004, \eta _p^2 = 0.143$) were significant, whereas the main effect for AR condition was not ($p = 0.388$). A further simple effect analysis revealed that the main effect for test time was significant without AR (GGe = 0.718, $F(1.44,40.18) = 66.627, p < 0.001, \eta _p^2 = 0.704$), whereas it was not significant with AR (GGe = 0.503, $p = 0.560$). Then, a post-hoc multiple comparison (Tukey’s test) was conducted for the condition without AR. We found that the score obtained prior to class was significantly smaller than that obtained right after class (difference = $-1.697$, $z = -11.471, p < 0.001$) and that obtained 1-week after class (difference = $-1.014$, $z = -6.855, p < 0.001$), indicating the effect of PPT-based teaching; the score obtained right after class was significantly larger than that obtained 1-week after class (difference = 0.683, $z = 4.616, p < 0.001$), indicating the power of forgetting. However, the effect of AR-based teaching was absent.

For the grade 1 and female students (bottom-left panel of Fig. 1, GGe = 0.695), the main effect for test time ($F(1.39,44.50) = 45.635, p < 0.001, \eta _p^2 = 0.588$) and the interaction effect ($F(1.39,44.50) = 6.684, p = 0.007, \eta _p^2 = 0.173$) were significant, whereas the main effect for AR condition was not ($p = 0.889$). A further simple effect analysis revealed that the main effects for test time were significant without AR (GGe = 0.920, $F(2,30) = 31.867, p < 0.001, \eta _p^2 = 0.680$) and with AR (GGe = 0.522, $F(1.04,17.76) = 25.736, p < 0.001, \eta _p^2 = 0.602$). Then, a post-hoc multiple comparison (Tukey’s test) was conducted for two AR conditions separately. Under the condition without AR, we found that the score obtained prior to class was significantly smaller than that obtained right after class (difference = $-1.688$, $z = -7.981, p < 0.001$) and that obtained 1-week after class (difference = $-0.875$, $z = -4.139, p < 0.001$); the score obtained right after class was significantly larger than that obtained 1-week after class (difference = 0.813, $z = 3.843, p < 0.001$). Under the condition with AR, we found that the score obtained prior to class was significantly smaller than that obtained right after class (difference = $-1.822$, $z = -5.691, p < 0.001$), and the score obtained right after class was significantly larger than that obtained 1-week after class (difference = 2.122, $z = 6.628, p < 0.001$); however, the score obtained 1-week after class was comparable with that obtained prior to class ($p = 0.617$). The results indicated the effects of PPT-based teaching and AR-based teaching. Moreover, students lost few scores 1-week after PPT-based teaching than AR-based teaching, indicating the effect of AR-based teaching being less persistent.

For the grade 2 and male students (top-right panel of Fig. 1, GGe = 0.951), the main effects for test time ($F(2,154) = 33.203, p < 0.001, \eta _p^2 = 0.301$) and AR condition ($F(1,77) = 5.237, p = 0.025, \eta _p^2 = 0.064$) were significant, and the interaction effect was marginally significant ($F(2,154) = 2.879, p = 0.059, \eta _p^2 = 0.036$). A further simple effect analysis revealed that the main effects for test time were significant without AR (GGe = 0.648, $F(1.30,36.31) = 12.641, p < 0.001, \eta _p^2 = 0.311$) and with AR (GGe = 0.596, $F(1.19,58.45) = 26.440, p < 0.001, \eta _p^2 = 0.350$). Then, a post-hoc multiple comparison (Tukey’s test) was conducted for two AR conditions separately. Irrespective of the AR condition, we found that the score obtained prior to class was significantly smaller than that obtained right after class (without AR: difference = $-0.972$, $z = -4.869, p < 0.001$; with AR: difference = $-0.676$, $z = -4.933, p < 0.001$), and the score obtained right after class was significantly larger than that obtained 1-week after class (without AR: difference = 0.703, $z = 3.522, p = 0.001$; with AR: difference = 0.972, $z = 7.093, p < 0.001$); however, the score obtained 1-week after class was comparable with that obtained prior to class (without AR: $p = 0.369$; with AR: $p = 0.078$). The results indicated the effects of PPT-based teaching and AR-based teaching and the power of forgetting. Moreover, due to the marginally significant interaction effect, students obtained larger scores and lost few scores 1-week after PPT-based teaching than AR-based teaching, indicating higher learning efficiency for PPT-based teaching than AR-based teaching.

For the grade 2 and female students (bottom-right panel of Fig. 1, GGe = 0.948), the main effect for test time ($F(2,76) = 17.171, p < 0.001, \eta _p^2 = 0.311$) and the interaction effect ($F(2,76) = 10.061, p < 0.001, \eta _p^2 = 0.209$) were significant, whereas the main effect for AR condition was not ($p = 0.146$). A further simple effect analysis revealed that the main effect for test time was significant without AR (GGe = 0.887, $F(2,50) = 37.085, p < 0.001, \eta _p^2 = 0.597$), whereas it was not significant with AR (GGe = 0.624, $p = 0.072$). Then, a post-hoc multiple comparison (Tukey’s test) was conducted for two AR conditions separately. Under the condition without AR, we found that the score obtained prior to class was significantly smaller than that obtained right after class (difference = $-1.577$, $z = -8.575, p < 0.001$) and that obtained 1-week after class (difference = $-0.915$, $z = -4.978, p < 0.001$); the score obtained after class was significantly larger than that obtained 1-week after class (difference = 0.662, $z = 3.597, p < 0.001$). Under the condition with AR, we found that the score obtained right after class was significantly larger than that obtained 1-week after class (difference = 0.857, $z = 2.631, p = 0.023$); however, the score obtained prior to class was comparable with that obtained right after class ($p = 0.544$) and that obtained 1-week after class ($p = 0.255$). The results indicated the effect of PPT-based teaching and the power of forgetting. However, students learned nothing from AR-based teaching and even performed worse 1-week later.

The separate analyses indicated that the utilization of AR technology in Chinese vocational colleges affected the academic performance of students of different sexes and grades differently. The results showed the absence of AR enhancement for the grade 1 and male students, less persistent AR enhancement for the grade 1 and female students, limited learning efficiency of AR-based teaching for the grade 2 and male students, and even AR detriment for the grade 2 and female students. Our hypothesis was not supported by the results. The two questions raised in the introduction part could not be answered easily. It was much more complicated.

### Studying effect, forgetting effect, and retaining effect of students

To get a more intuitive understanding of our results, we defined three parameters as follows: studying effect (SE), that is, the score obtained right after class minus the score obtained prior to class; forgetting effect (FE), that is, the score obtained right after class minus the score obtained 1-week after class; retaining effect (RE), that is, the score obtained 1-week after class minus the score obtained prior to class. We performed a two-way mixed ANOVA, with sex (male and female), grade (grade 1 and grade 2), and AR condition (with AR and without AR) regarded as the between-subject factor for SE, FE, and RE, respectively.

**Figure 2:**
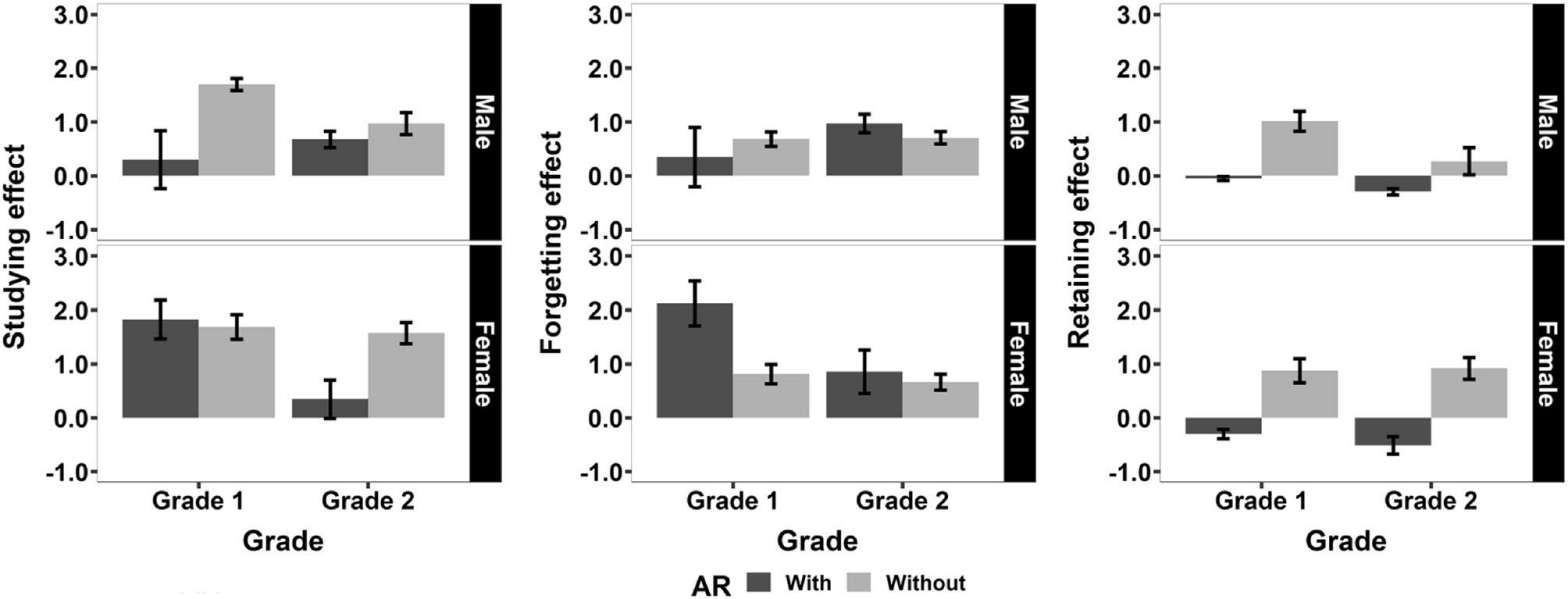
The results of SE, FE, and RE. Within each panel, the abscissa represents grade, and the ordinate represents SE, FE, and RE, respectively. The color of the bars represents whether the students learned with AR or not. The error bars represent the standard error of the means across students.

As shown in Fig. 2, regarding SE, we found that female students had significantly higher SE than male students ($F(1,190) = 5.889, p = 0.016, \eta _p^2 = 0.030$), grade 1 students had significantly higher SE than grade 2 students ($F(1,190) = 6.946, p = 0.009, \eta _p^2 = 0.035$), students learned with AR had significant lower SE than students learned without AR ($F(1,190) = 14.419, p < 0.001, \eta _p^2 = 0.071$). Also, the interaction effect among sex, grade, and AR condition was significant ($F(1,190) = 11.273, p < 0.001, \eta _p^2 = 0.056$). The remaining effects were insignificant ($p > 0.05$). Regarding FE, we found that female students had significantly higher FE than male students ($F(1,190) = 5.541, p = 0.020, \eta _p^2 = 0.028$), students learned with AR had marginally significantly higher FE than students learned without AR ($F(1,190) = 3.779, p = 0.053, \eta _p^2 = 0.019$). Also, the interaction effects between sex and grade ($F(1,190) = 7.711, p = 0.006, , \eta _p^2 = 0.039$), between sex and AR condition ($F(1,190) = 4.482, p = 0.036, , \eta _p^2 = 0.023$), and among three factors ($F(1,190) = 5.354, p = 0.022,, \eta _p^2 = 0.027$) were significant. The remaining effects were insignificant ($p > 0.05$). Regarding RE, we found that grade 1 students had significantly higher RE than grade 2 students ($F(1,190) = 4.969, p = 0.027, \eta _p^2 = 0.025$), students learned with AR had significantly lower RE than students learned without AR ($F(1,190) = 65.646, p < 0.001, \eta _p^2 = 0.257$). The remaining effects were insignificant ($p > 0.05$). The descriptive results of SE, FE, and RE for students with different sexes and grades are summarized in Table 2.

**Table 2:** The descriptive results of SE, FE, and RE for students with different sexes and grades. The standard errors are placed in parentheses.

	Studying effect		Forgetting effect		Retaining effect	
	Grade 1	Grade 2	Grade 1	Grade 2	Grade 1	Grade 2
Male						
With	0.300 (0.540)	0.676 (0.152)	0.350 (0.554)	0.972 (0.172)	− 0.050 (0.039)	− 0.296 (0.061)
Without	1.697 (0.112)	0.972 (0.206)	0.683 (0.133)	0.703 (0.116)	1.014 (0.188)	0.269 (0.253)
Female						
With	1.822 (0.359)	0.343 (0.357)	2.122 (0.414)	0.857 (0.406)	− 0.300 (0.087)	− 0.514 (0.163)
Without	1.688 (0.227)	1.577 (0.195)	0.813 (0.177)	0.662 (0.148)	0.875 (0.226)	0.915 (0.204)

Then, to further examine the interaction effects we reported before, we performed the two-sided independent Welch t-test for students with different sexes and grades to compare the differences of SE, FE, and RE, respectively, between conditions with AR and without AR. The statistical results are depicted in Tables 3 and 4. Consistent with the conclusions drawn from ANOVA analysis, a better academic performance would be accomplished with PPT-based teaching. Regarding SE, grade 1 and male students and grade 2 and female students would benefit more from PPT-based teaching than AR-based teaching. Regarding FE, the two teachings had almost no difference except for grade 1 and female students, who would forget much more knowledge learned with AR than without AR. Regarding RE, all students could retain much more knowledge they learned without AR than with AR. Moreover, the negative RE for all students who had learned with AR implied the adverse effect of AR-based teaching.

**Table 3:** The statistical results (Welch t-test) for two AR conditions.

	Studying effect		Forgetting effect		Retaining effect	
	Grade 1	Grade 2	Grade 1	Grade 2	Grade 1	Grade 2
Male						
*t*	− 2.532	− 1.159	− 0.584	1.298	− 5.548	− 2.169
*df*	16.305	57.27	16.757	76.133	30.330	31.324
*p*	0.022 (*)	0.251	0.567	0.198	< 0.001 (***)	0.038 (*)
Female						
*t*	0.318	− 3.035	2.909	0.453	− 4.840	− 5.475
*df*	28.189	20.968	22.947	16.534	19.425	37.594
*p*	0.753	0.006 (**)	0.008 (**)	0.656	< 0.001 (***)	< 0.001 (***)

**Table 4:** The means and differences of SE, FE, and RE for two AR conditions.

	Studying effect		Forgetting effect		Retaining effect	
	Grade 1	Grade 2	Grade 1	Grade 2	Grade 1	Grade 2
Male						
With AR	0.300	0.676	0.350	0.972	− 0.050	− 0.296
Without AR	1.697	0.972	0.683	0.703	1.014	0.269
Difference	− 1.397 (*)	− 0.296	− 0.333	0.269	− 1.064 (***)	− 0.565 (*)
Female						
With AR	1.822	0.343	2.122	0.857	− 0.300	− 0.514
Without AR	1.688	1.577	0.813	0.662	0.875	0.915
Difference	0.135	− 1.234 (**)	1.310 (**)	0.196	− 1.175 (***)	− 1.430 (***)

## Discussion

We examined whether students in a Chinese vocational college with different sexes and grades could benefit from AR-based teaching. We distributed the students into the PPT-based group and AR-based group. The scores were obtained from each student at three test times: prior to class, right after class, and 1-week after class. We found that academic performance was significantly improved through PPT-based teaching (without AR) for students of different sexes and grades. On the other hand, our results implied the inefficiency of AR-based teaching (with AR). The grade 1 and male students and grade 2 and female students could not even improve their scores right after class with the aid of AR. For grade 1 and female students who learned with AR, their long-lasting memories were inferior to those who learned without AR. 1-week later, all students, irrespective of sex and grade, performed better if they underwent PPT-based teaching than AR-based teaching.

We also analyzed the studying effect (SE), forgetting effect (FE), and retaining effect (RE) for students with different sexes and grades. Firstly, female students had higher SE than male students. This was consistent with the female advantage in the educational field reported in many previous studies^38–40^. Female students were more likely to have better academic performance than male students. Secondly, grade 1 students had higher SE and RE than grade 2 students. We presumed that the poor performance of grade 2 students was caused by the sophomore slump, a phenomenon depicting the decrement in academic performance for grade 2 students^41,42^. Grade 2 students gradually lost the feeling of freshness for the environment, received less attention from teachers, and felt stronger academic pressure and career confusion, leading to the sophomore slump.

Thirdly, students who learned with AR had lower SE and RE, whereas higher FE than those who learned without AR. In other words, we reported the inefficiency of AR-based teaching, especially for RE. AR could even elicit worse performance 1-week after class. This was inconsistent with most of the previous studies, wherein researchers reported the positive effect of AR on education^2,5,8,14,17^. We presumed that the different identities of the recruited students between the current study and previous studies might be one possible reason. Previous studies recruited K-12 and higher education students, whereas the current study recruited students from vocational colleges^14^. Due to the peculiar conditions of China, students in Chinese vocational colleges have worse learning abilities and academic performance than other students. Radu^5^ summarized why students might perform worse with AR than without AR. Students might be overwhelmed when manipulating 3D material and listening to the teacher in the meantime; students could get distracted and focus only on the 3D material while ignoring what the teachers taught; the class discipline could be more chaotic because of the use of AR applications on personal mobile phones. Therefore, deploying AR would negatively affect students’ performances, especially for students with less self-discipline ability.

Another possible reason might be attributed to the difference in the teaching material and way of examination between the current study and previous studies. Previous studies mainly used abstract concepts, such as the solar system, molecular movement, and physics law, as the teaching material, whereas the current study used a concrete 3D material as the teaching material^5,14^. As to the way of examination, previous studies evaluated the affective states, such as motivation, engagement, and satisfaction, rather than the direct test of what students had learned^2,5,14^. Furthermore, if the understanding of conceptual knowledge was used to evaluate the effect of AR technology, no significant learning gains would be reported between the AR group and control group^43,44^. Conversely, in the current study, students were taught the three views of a 3D material and asked to draw the three views as the examination. The consistency of teaching material and examination could provide direct evidence of to what extent students had mastered the key knowledge they learned. Although students could better understand three views of the 3D material with AR, the examination was in the way of 2D drawing. Therefore, students who learned without AR had seen the 2D version during the PPT-based teaching, which might have helped them obtain higher scores in the examination.

Intriguingly, there was a significant interaction effect between sex and grade. For grade 1 and female students and grade 2 and male students, they could obtain comparable scores when they learned with AR and without AR, whereas for grade 1 and male students and grade 2 and female students, they could obtain larger scores when they learned without AR than with AR. We could not find the possible reasons for the interaction effect, and further study was needed to verify such an effect.

Previous studies have largely endorsed the use of new technologies in educational processes. Södervik et al.^43^ reported that pharmacy students in research universities under AR-assisted learning condition could do better laboratory work (antimicrobial susceptibility testing) compared to students under traditional learning condition. Even in mathematical education, the utilization of AR technology during the class could result in better learning performance compared to traditional classroom^45,46^. However, the unique characteristics of the students involved were usually overlooked. The current study, in its focus on students from vocational colleges, discovered that the use of AR can, in fact, impede students’ grasp of knowledge. Students from vocational colleges typically have lower learning abilities than their counterparts at research universities. Vocational education generally hinges more on practicality and application, with less emphasis on theoretical foundations. Additionally, within the sociocultural context of China, persistent neglect of vocational-technical education and its association with lower-income families exacerbate the scarcity of resources and opportunities available to students from vocational colleges. Over-reliance on AR can have negative implications for students with certain learning styles. For instance, students who excel at or prefer traditional learning methods may find AR distracting. Similarly, students with a lower tech-savviness may find AR overly complicated, which could interfere with their productivity. Therefore, when contemplating the integration of novel technology in the classroom, educators must judiciously consider individual student differences to ensure equal benefit. The current study underscores this argument, revealing that AR doesn’t tend to yield significant benefits for students from vocational colleges. In our continuous effort to update educational technologies, the individual capabilities of students-the recipients of these innovations-must be taken into account. Otherwise, the outright pursuit of advancements can paradoxically yield poorer outcomes. Our study also uncovered the significant interaction effect between students’ sexes and grades (not age). Research universities primarily follow a four- or five-year system, and students often remain in the educational space to obtain a postgraduate degree for added competitiveness. Contrarily, vocational schools typically have a three-year system with highly applied instructional objectives, and their students rarely pursue advanced studies, transitioning instead directly into the workforce. These societal exigencies exacerbate the effect of sex and grade on the learning outcomes of students from vocational colleges, a fact confirmed by the current study.

Consequently, based on our results, we would like to make several pedagogical recommendations. First, although students’ motivation, satisfaction, and engagement might be enhanced with AR, the direct consequence (scores of class examinations) might not be satisfying for the teachers. It should be well-considered whether it is necessary to use AR during the class. Second, AR could be effective or not. It depends on the sex and grade of the students. Teachers could decide whether to use AR during the class, depending on the receiver of the class. Third, there are female advantages and sophomore slumps for students in vocational colleges. Teachers could pay more attention to students with poor performance. Furthermore, when limited to the teaching of the Engineering Drawing course in vocational colleges, we suggested that teachers could follow the traditional PPT-based teaching rather than the novel AR-based teaching. However, we could not wholly disregard the possible positive effects of AR technology reported by previous studies. Although it might not be a good idea to apply AR technology solely, the combination of PPT-based teaching and AR-based teaching might improve students’ academic performance. Further studies are needed.

There were several limitations in the current study. First, students in this study were from one department at one university. It remained unclear whether the results obtained in this study could be generalized to other departments or other vocational colleges. It is better to recruit students from different departments and vocational colleges in the future to reproduce the current study. Second, during the second stage of the experiment, students in the AR-based group executed the AR application simultaneously. The Internet lag sometimes occurred, resulting in a less smooth experience of AR-based teaching. Due to the limitations of communication technology, it is difficult to solve the Internet problem. Rather, it is better to distribute students in the AR-based group into two or more subgroups and conduct the experiment at a separate time. Nevertheless, we reported the inefficiency of AR for students in vocational colleges. Students performed worse when learning with AR than when learning without AR. Although AR could be effective for specific students, the efficiency was comparable with students learning without AR. Teachers should not put too much faith in the effects of AR.

## Methods

### Participants

Through the instruction from two of the authors (representatives of secondary colleges and those in charge of student affairs), the student union organized educational experiments and recruited 211 first and second-year students (age range, 18–20 years old) to participate in the experiment. All students majored in architecture from the Jiujiang Vocational and Technical College. Thirteen students failed to follow our instructions and were eliminated from further analysis, leading to a total of 198 students. Of them, there were 45 grade 1 and male students, 79 grade 2 and male students, 34 grade 1 and female students, and 40 grade 2 and female students. The students were further distributed into two groups: PPT-based and AR-based. The group distribution was completely randomized, with a balanced number of participants in each group. As a result, there were 100 students in the PPT-based group, including 29 grade 1 and male students, 16 grade 1 and female students, 29 grade 2 and male students, and 26 grade 2 and female students. There were 98 students in the AR-based group, including 16 grade 1 and male students, 18 grade 1 and female students, 50 grade 2 and male students, and 14 grade 2 and female students.

We confirmed that informed consent was obtained from all students and/or their legal guardian(s). We confirmed that all methods were carried out in accordance with relevant guidelines and regulations. We confirmed that all experimental protocols were approved by the Ethics Committee of Jiujiang Vocational and Technical College.

### Apparatuses

**Figure 3:**
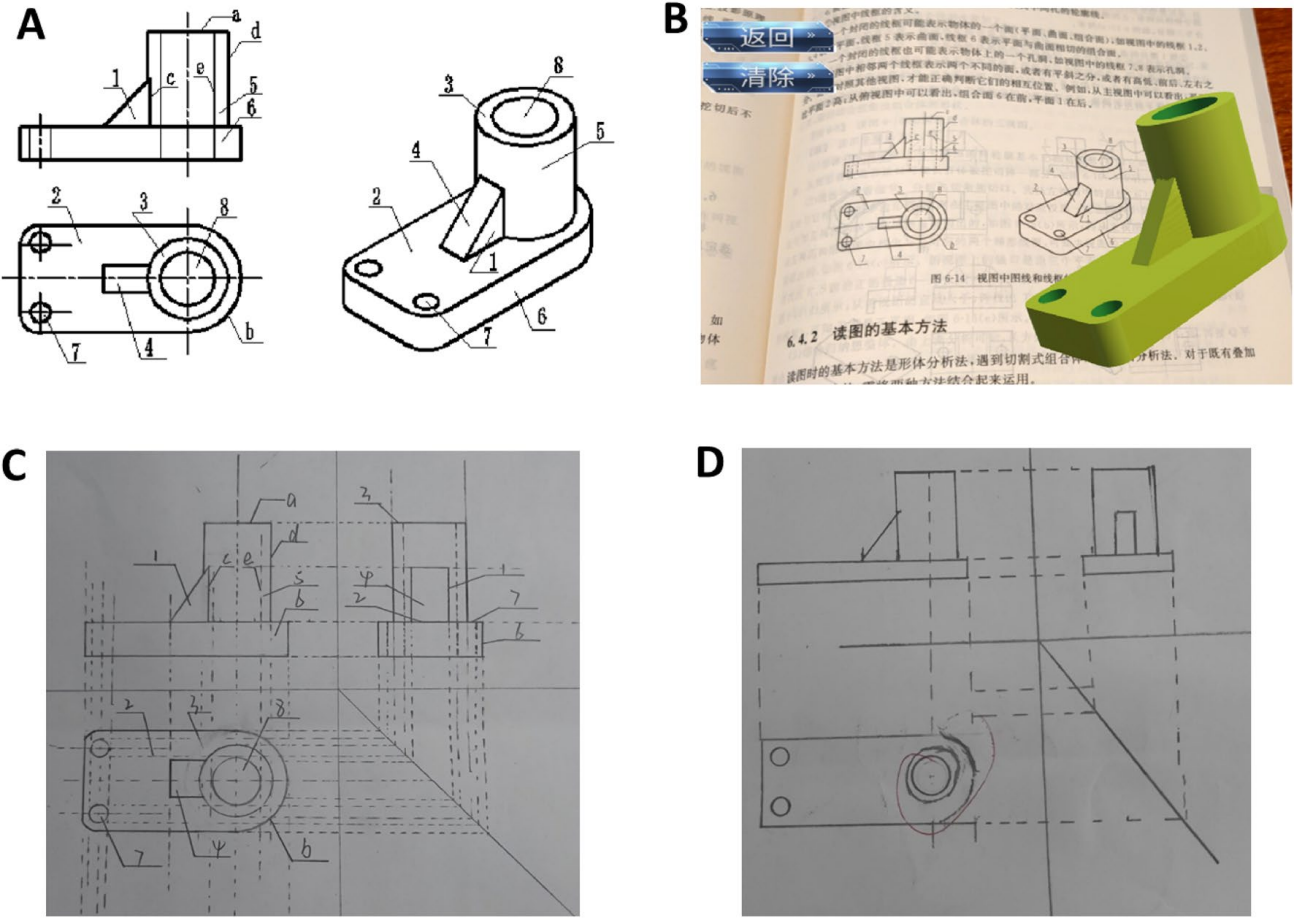
The illustrations for teaching material and examples of students’ examination papers. (**A**) The standard illustrations of the side view and top view of the material; (**B**) the AR illustration on the mobile phone; (**C**) an example of a student’s examination paper with a score of 18 points; (**D**) an example of a student’s examination paper with a score of 14 points.

Each recruited student should hold their mobile phone as the AR apparatus. We used a book entitled “Understanding of Architectural Graphics” as the teaching material (please refer to Fig. 3A). Students could download an AR application by scanning the two-dimensional code attached to the book to watch the three-dimensional (3D) modeling materials designed specifically for the chosen book (Fig. 3B). The students could zoom in, zoom out, split, or merge the 3D material through their finger manipulations.

### Procedures

The experiment consisted of three stages. In the first stage (prior to class), students from PPT-based and AR-based groups were asked to read the teaching material. Fifteen minutes later, they were asked to draw the frontal view, side view, and top view of a 3D material within another 15 min to assess their existing drafting skills. During the class, for the students in the PPT-based group, the teacher presented a 15-min lecture on the relationships and drawing rules of the orthographic projections, using a PowerPoint presentation for clarity; on the other hand, for the students in the AR-based group, they used professional mobile applications to scan QR codes in textbooks, generating Augmented Reality (AR) three-dimensional diagrams. The teacher further explained for 15 min, using these generated AR images as teaching materials. Afterward, in the second stage (right after class), the students from two groups were given another 15 min to redraw the diagrams. In the third stage (1-week after class), that is, a week later, the same group of students was recruited to redraw the same engineering diagram within a 15-min window for a final time. This stage served to measure students’ progress and analyze the effect of different teaching methods.

To ensure that the effectiveness of classroom instruction is not influenced by the varying teaching abilities of different teachers, we invited only one teacher who is currently teaching this course in the frontline to guide the experimental process. This strategy helps maintain consistent teaching quality and provides students with a stable learning experience. As a result, the experiment for the students in different groups during the class was conducted on separate days.

As to the rating criteria, the maximum score of the examination was 20 points: 5 points for the overall aesthetic, 5 points for the frontal view, 5 points for the side view, 5 points for the top view, and 5 points for the correspondence among the three views. More specifically, one point for completeness, which confirmed whether all necessary information and critical details of the object were accurately depicted; one point for clarity, which checked if all the details and dimensions were clearly visible; one point for accuracy, which verified whether all measurements and scales were precise; one point for standardized symbols, which checked whether the drawing followed standard engineering symbols and terminology; one point for readability, which inspected whether the layout of the drawing was easily comprehensible. This is an objective criterion for rating the understanding of an orthographic projection. It is widely adopted for the Engineering Drawing course. Therefore, the validity should be confirmed. Considering the rating preferences of different teachers, we recruited only one teacher to rate all the examination papers. In the current study, the teacher who rated the examination papers was the person who taught the lesson. In so doing, all the examination papers could be rated based on the explicit rating criteria. Furthermore, to ensure the reliability of ratings, we use a mode called inter-rater reliability. We selected a portion of the examination papers, asked the teacher who instructed the class, and rated the papers to explain his grading approach to three other teachers. If the three teachers disagreed with the final marks, the paper in question must be regraded. The results showed that the rating teacher had a clear grading approach, and the other teachers agreed upon all the selected test papers’ scores. There were three examinations during the experiment, leading to three scores at three test times for each student: prior to class, right after class, and 1-week after class. Please refer to Fig. 3C,D for examples of students’ examination papers. The score for Fig. 3C was 18 points, and that for Fig. 3D was 14 points.

## Data Availability

The datasets generated and/or analyzed during the current study are available in the OSF repository (https://osf.io/v6aqt/).
